# An Alternative to Dye-Based Approaches to Remove Background Autofluorescence From Primate Brain Tissue

**DOI:** 10.3389/fnana.2019.00073

**Published:** 2019-07-18

**Authors:** Wonn S. Pyon, Daniel T. Gray, Carol A. Barnes

**Affiliations:** ^1^Evelyn F. McKnight Brain Institute, The University of Arizona, Tucson, AZ, United States; ^2^ARL Division of Neural Systems, Memory and Aging, The University of Arizona, Tucson, AZ, United States; ^3^Department of Psychology, Neurology and Neuroscience, The University of Arizona, Tucson, AZ, United States

**Keywords:** confocal imaging, autofluorescence, Sudan Black B, spectral imaging, linear unmixing, immunohistochemistry, fluorescence microscopy

## Abstract

Brain tissue contains autofluorescing elements that potentially impede accurate identification of neurons when visualized with fluorescent microscopy. Age-related accumulation of molecules with autofluorescent properties, such as lipofuscin, can possess spectral profiles that invade the typical emission range of fluorophores commonly utilized in fluorescent microscopy. The traditional method for accounting for this native fluorescence is to apply lipophilic dyes that are able to sequester these unwanted signals. While effective, such dyes can present a range of problems including the obstruction of fluorescent probe emissions. The present study utilizes aged primate midbrain tissue stained for tyrosine hydroxylase and calbindin to investigate an image processing approach for removing autofluorescence utilizing spectral imaging and linear unmixing. This technique is then compared against the traditional, dye-based autofluorescence sequestration method using Sudan Black B (SBB). Spectral imaging and linear unmixing yielded significantly higher cell numbers than SBB treatment. This finding suggests that computational approaches for removing autofluorescence in neural tissue are both viable and preferential to dye-based approaches for estimation of cell body numbers.

## Introduction

Fluorescence microscopy has become a critical tool in the biological sciences for visualizing different cellular and subcellular components within a single section of tissue, providing more information than more traditional forms of light microscopy. The ability to label multiple elements of a single sample has been especially powerful for investigating the chemical anatomy of the nervous system, revealing the striking heterogeneity in the genetic makeup of different neurons and glial cells. Brain tissue, however, is littered with various autofluorescing elements that can reduce the quality of the images obtained with this method. Factors known to induce autofluorescence include products of chemical tissue fixation (Clancy and Cauller, 1998), intrinsic tissue components such as vasculature (Edwin and Jackman, 1981; Mansfield et al., 2005) and lysosomal aggregates as a result of natural aging (Belichenko et al., 1996). One of the more common accumulations found in aged neural tissue exists in the form of lipofuscin, a collection of various lipids and trace metals within lysosomes that evades degradation via normal cellular mechanisms (for review, Terman and Brunk, 2004). Lipofuscin presents itself as punctate yellow-gold artifacts that can amass to surround the entirety of a neuron’s cell body (Brizzee et al., 1974). It has been shown to possess wide fluorescence excitation and emission ranges that invade the emission spectra of commonly used fluorophores utilized in fluorescent microscopy (Eldred and Katz, 1988; Feldman et al., 2015). Consequently, studies utilizing fluorescent imaging techniques to quantify the expression of different chemical components of aged neural tissue are often confounded by false-positives introduced from native fluorescence of lipofuscin.

Various treatment methods have been utilized for combatting autofluorescence. These include chemical treatments using ferrous chloride, ferric chloride, cupric sulfate (Kikugawa et al., 1997), cupric chloride (Schnell et al., 1999), and photobleaching the unwanted fluorescence (Schnell et al., 1999). The most popular method, however, involves the sequestration of fluorescent emission with lipophilic dyes such as Sudan Black B (SBB) (Meister et al., 1991; Romijn et al., 1999). While effective for combatting lipid-based fluorescence, this treatment has a number of undesirable side-effects. For example, all treated tissue is permanently dyed a deep blue and the dye itself has been shown to fluoresce at deep red-shifted wavelengths (Diez-Fraile et al., 2012). Furthermore, SBB-treatment can reduce and sometimes even completely obscure the emission of fluorescent probes used in immunohistochemical experiments, consequently removing signal and negatively effecting stereological experiments (Schnell et al., 1999). Limitations such as these highlight the importance of developing systematic and reliable methods for the removal of native fluorescence, particularly when studying aged neural tissue.

The present study investigates a non-chemical, imaging-based approach to autofluorescence removal in neural tissue through an image acquisition and processing protocol known as spectral imaging and linear unmixing. In fluorescence microscopy, the standard and most common imaging techniques involve the recording of pixel intensity values based on the photoemission of fluorophores within a defined collection window (Lichtman and Conchello, 2005). In spectral imaging, the spectral profile of each individual pixel is recorded and can be utilized for image processing (Zimmermann et al., 2003). Assuming a given pixel’s spectral profile is the linear mixture of a number of emitting elements, multispectral pixels can be deconvolved based on the relative contribution of reference spectra for elements known to be present within an image (Dickinson et al., 2001; Lansford et al., 2001; Mansfield et al., 2005; Zimmermann et al., 2014). This process is known as linear unmixing.

Spectral imaging and linear unmixing has already been successfully used to distinguish multiple fluorophores that emit at similar wavelengths (Lansford et al., 2001; Zimmermann et al., 2003). It also can decipher the relative contribution of spatially overlapping fluorophores (Guzowski et al., 2005; Aswendt et al., 2019), as well as parse out fluorophore signals from unwanted background emissions (Mansfield et al., 2005; Mylle et al., 2013; Yamanaka et al., 2018). How spectral imaging and linear unmixing compares to previously utilized methods of autofluorescence removal in neural tissue has not been investigated.

The goal of this study was to investigate the viability of spectral imaging and linear unmixing as an alternative to SBB treatment for the removal of autofluorescence in aged neural tissue. Since autofluorescence removal techniques are particularly necessary for regions of heavy autofluorescence, the experiments reported here targeted dopaminergic- and calbindin-expressing neurons of the midbrain as this region has been reported to show heavy lipofuscin accumulation in aged animals (Brizzee et al., 1974).

## Materials and Methods

### Subjects

The tissue used in the present study was collected from 4 aged rhesus macaques, *Macaca mulatta* [26.3 (M), 28.2 (M), 28.3 (F), and 31.1 (F) years] while the single-label control hemisections were taken from 2 young rhesus macaques [13.4 (M) and 11.2 (M)]. Maximal lifespan estimates for humans and *M. mulatta* have been used to estimate the equivalent human age of the non-human primate compared to humans (Ershler et al., 1988; Tigges et al., 1988; Hakeem et al., 1996). To calculate the equivalent human age of the macaque, a multiplication factor of 3 has been suggested. Thus these animals would be considered to be the equivalent of 69–93 human years. All animals were paired housed at the California National Primate Research Center in Davis, CA, United States and participated in a long-term behavioral and electrophysiological study. This study was carried out in accordance with guidelines stipulated in the Guide for the Care and Use of Laboratory Animals by the National Institutes of Health and all of the described protocols were reviewed and approved by the Institutional Animal Care and Use Committee at the University of California, Davis.

### Perfusion and Immunohistochemistry

**Figure 1:**
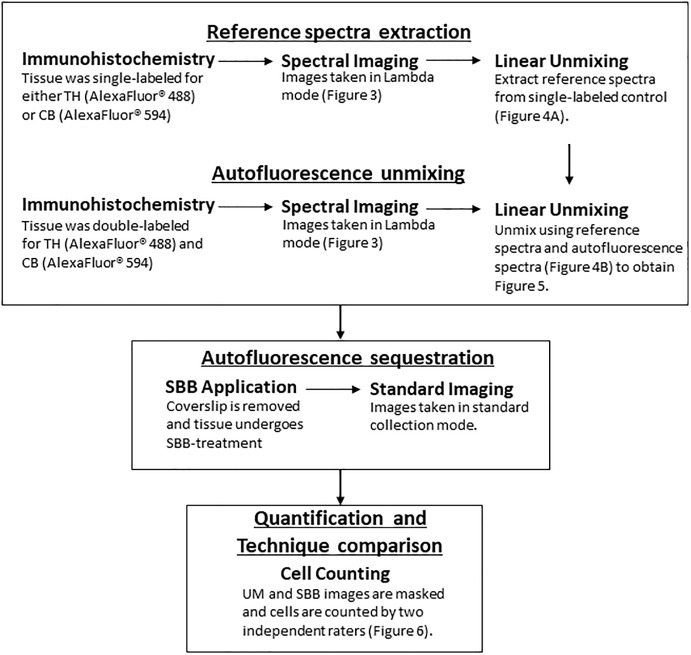
A flowchart depicting the workflow (TH, Tyrosine Hydroxylase; CB, Calbindin; UM, Unmixed; SBB, Sudan Black B).

A schematic of the workflow can be found in Figure 1. Animals were anesthetized with an overdose of sodium pentobarbital (60 mg/kg, i.v.), and transcardially perfused with a solution of 4% paraformaldehyde (PFA) in 0.01M phosphate buffer saline (PBS; pH 7.4) followed by a solution of 4% PFA and 10% sucrose. Brains were extracted and left to float freely in 4% PFA and 30% sucrose at 4°C until the brains became saturated and sank. The brains were then cut coronally into 30 μm sections on a freezing microtome and stored long-term in O.C.T. at -80°C.

The regions of interest for the present study included the ventral tegmental area (VTA), retrorubral field (RRF), and the substantia nigra (SN). These dopamine neuron-rich regions (Fuxe, 1965; Kanaan et al., 2007) were identified based on descriptions found in the Paxinos et al., 2000, Rhesus Monkey Atlas and through visible tyrosine hydroxylase-expression.

Tissue was thawed, hemisected and washed in PBS (3 × 5 min). Following these washes, the tissue underwent a water-bath heated antigen retrieval regimen in which each section free-floated for 30 min in sodium citrate buffer (pH 9) that was heated to 80°C. The buffer solution was then allowed to return to room temperature after which the tissue was removed and washed again in PBS (3 × 5 min). To minimalize non-specific antibody binding, the tissue underwent a 1 h block incubation at room temperature in 10% normal donkey serum (NDS; Sigma-Aldrich, D9663-10ML) with 0.25% Triton^TM^ X-100 (TX; Sigma-Aldrich, x100) added to permeabilize the tissue. The tissue was incubated with primary antibodies for tyrosine hydroxylase (polyclonal anti-TH in Rabbit, 1:1000; Abcam, ab137721) and the calcium-binding protein, calbindin (monoclonal anti-CB in Mouse, 3:2000; Abcam, ab75524), diluted in 10% NDS with 0.04% TX for 48 h at 4°C. The tissue was then washed in PBS (4 × 10 min) and incubated for 2 h at room temperature in a solution with two secondary antibodies, one conjugated with Alexa Fluor^®^ 488 (peak emission: 519 nm, donkey anti-rabbit, 4:2000; Abcam, ab150073) and another conjugated with Alexa Fluor^®^ 594 (peak emission: 617 nm, donkey anti-mouse, 5:2000; Abcam, ab150108) diluted with 10% NDS. After a final wash in PBS (5, 15, and 30 min), the sections were mounted on 1.0 mm thick plus slides (Brain Research Laboratories, #5075-PLUS). The sections were coverslipped using 80% glycerol and PBS mounting medium and a #1.5 thickness coverslip (Brain Research Laboratories, #4860-1½, 0.16 – 0.19 mm thick) and sealed with nail polish.

Two sections corresponding to rostral and caudal midbrain [approximately Paxinos plate 67 and 82, Bregma -11.25 mm, and -18.00 mm, respectively (Paxinos et al., 2000)] were taken from each of the four brains (16 total hemisections). One rostral hemisection from a single brain was utilized as an unstained control. This control section underwent an identical incubation protocol with primary and secondary antibodies excluded from incubating solutions.

Two hemisections from two young macaques were utilized as single-label controls. Each hemisection underwent identical incubation procedures as previously stipulated but with only one antibody in the primary and secondary incubations (either tyrosine hydroxylase visualized with Alexa Fluor^®^ 488 or calbindin visualized with Alexa Fluor^®^ 594). Sections from young macaque brains were specifically utilized for the single-label control so as to minimize spectral interference with tissue autofluorescence.

### Spectral Imaging Through “Lambda” Collection (Pre-sudan Black B Treatment)

**Figure 2:**
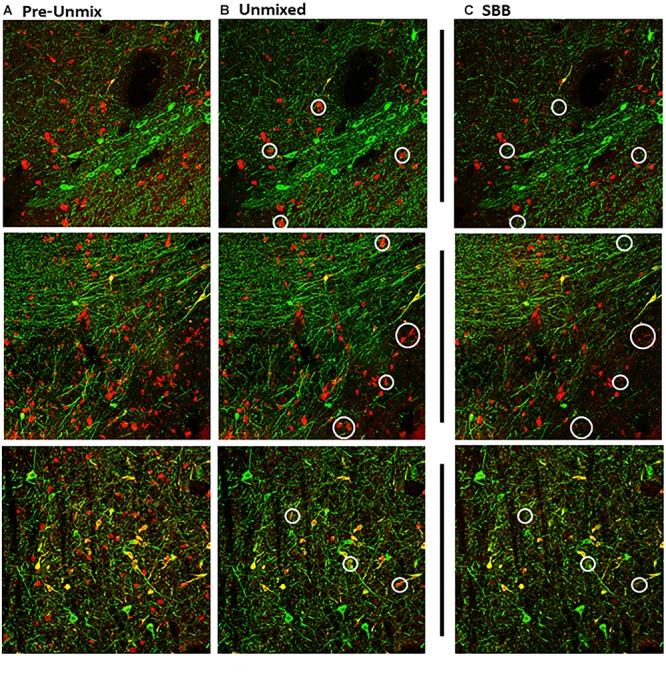
Maximum intensity projections of *z*-stack images taken from double-immunolabeled hemisections. **(A)** Original image prior to unmixing. ZEN Black categorizes pixels of the original image based on the relative contribution of the user-identified reference spectra. **(B)** Original image after the removal of pixels corresponding to the autofluorescence reference spectra (Green, Tyrosine Hydroxylase; Red, Calbindin D-28k). **(C)** Same hemisection but re-imaged after SBB treatment. Images shown in **(B,C)** reflect Unmixed and SBB images of similar FOVs taken on two different sessions. Note the distinct difference in signal between **B,C**, circled in white.

**Figure 3:**
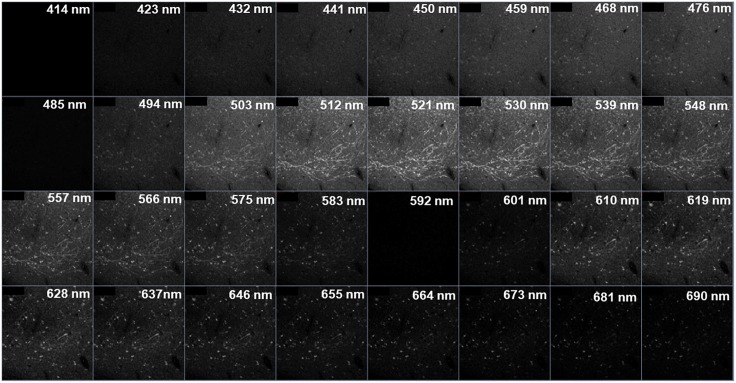
Visualization of a “Lambda” collected image for stained tissue. Each square represents a z-stack taken at a particular range of wavelength (e.g., “503 nm” denotes a *z*-stack collected from 499 to 507 nm). There are a total of thirty-two 8.9 nm bins. Note that the visible emissions for Alexa Fluor^®^ 488 are from ∼503–575 nm and Alexa Fluor^®^ 594 from ∼601–690 nm.

Z-stacks (1 μm steps, 8-bit, 2048 × 2048 resolution, pixel dwell of 4.12 μs, line average of 1) were imaged at a 708.5 μm by 708.5 μm field of view (FOV) through a Plan-Apochromat 20x/0.8 air objective using a ZEISS LSM 880 inverted confocal microscope. TH and CB were visualized using 488 nm (Argon, 25 mW at ∼0.7–1.2% power) and 594 nm (HeNe, 2 mW at ∼15–55% power) lasers, respectively. While no 405 nm fluorophore was used in the experiment, a 405 nm (Diode 405-30, 30 mW at ∼1–6% power) laser was used during this stage of image collection to aid in spectral characterization (Jung et al., 2010). Examples of FOVs prior to unmixing can be found in Figure 2A. The aforementioned laser lines were utilized simultaneously using the “Lambda” collection mode found on the ZEN Black 2.1 imaging software that allowed for the collection of pixel intensity data along the full spectrum of emission (410 – 695 nm) in thirty-two distinct, 8.9 nm wide bins (Figure 3). Photomultiplier tube (PMT) gain was set between 650 and 680 while digital gain was 0 and digital offset was 1.

No systematic sampling scheme was employed since the goal was not to obtain estimates of cell number or density for any particular nuclei, but rather to compare the signal within specific FOVs following each treatment. 2–6 images were taken from each of the 15 double-labeled hemisections resulting in a total of 72 images. The number of images taken per hemisection was dependent on the size of the midbrain and the extent of tyrosine hydroxylase-expression within the particular hemisection. At minimum, a single 708.5 μm by 708.5 μm image was taken in each TH-expressing nucleus (RRF, SN, hypothalamus, and the VTA). In sections where the nuclei were sufficiently large, a second image was taken.

### Spectral Characterization of Fluorescence

**Figure 4:**
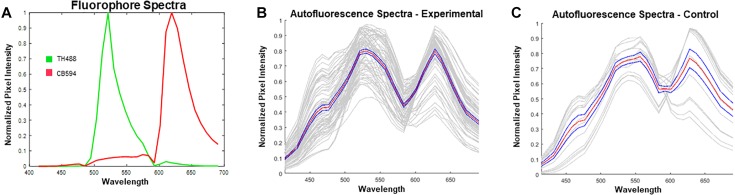
Reference spectra utilized prior to unmixing in ZEN Black. **(A)** Plotted in green is the emission spectrum for tyrosine hydroxylase visualized with Alexa Fluor 488^®^ (TH488). In red is the emission spectrum for calbindin visualized with Alexa Fluor^®^ 594 (CB594). **(B)** Plotted in gray are the autofound emission spectra for the autofluorescence (*n* = 72 images from 15 hemisections). Plotted in red is the average of the found autofluorescence spectra with standard error of the mean in blue. **(C)** Plotted in gray are the emission spectra for autofluorescence extracted from unstained, control tissue. Plotted in red is the average of the control autofluorescence spectra with standard error of the mean in blue (*n* = 14 images from 1 hemisection). Note the distinct dual-peak spectra (∼530 nm and ∼620 nm) found from the autofluorescence in both stained **(B)** and unstained tissue **(C)**. Furthermore, there exists a short wavelength (∼450 nm) emission in the autofluorescence spectra which enables further distinguishing of autofluorescence from antibody fluorescence.

Prior to unmixing the images in ZEN Black, reference spectra for Alexa Fluor^®^ 488 (excited by a 488 nm Argon laser) and 594 (excited by a 594 HeNe laser) were extracted from single-label control sections. These reference spectra were then uploaded into the software (Figure 4A). Given the spectra for the known fluorophores within the experimental tissue, the software was allowed to “Auto-Find” a third spectrum for autofluorescence, in the given image (Figure 4B). The spectral characteristics of the auto-found fluorescence were visually compared with spectra obtained from unstained control sections to ensure that the program had properly found the spectra for the autofluorescing element (Figure 4C). The matching auto-found spectra was then utilized as the reference spectrum for the autofluorescence for that particular image. This process was repeated for each individual image in order to account for potential differences in autofluorescence emission as a result of tissue age, perfusion efficacy, immunohistochemical treatment, or imaging that could alter the software’s ability to reliably identify and unmix the autofluorescence from the fluorophore label.

### Linear Unmixing

**Figure 5:**
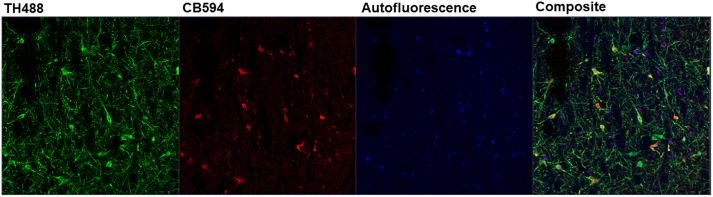
Depicted is the output of the ZEN Black software after linear unmixing an image. Three reference spectra were input into ZEN Black (e.g., “TH488” representing tyrosine hydroxylase, “CB594” representing calbindin and an autofluorescence spectra). The software uses these user-input reference spectra to categorize the pixels of the raw image and output three distinct channels corresponding to each reference spectra, with a fourth “Composite” channel created from merging the three.

Linear unmixing involves mathematically identifying the individual spectral contributions of various fluorescing elements in an image based on their known spectral characteristics. The linear unmixing feature in the ZEN Black software utilizes a least-squares fit based algorithm to separate pixels into distinct channels based on the contribution of that channel’s particular reference spectra (Mansfield et al., 2005; for review, Zimmermann et al., 2003). Up to ten unique reference spectra can be uploaded into this particular software program, allowing for a 10-channel image. For the present study, three reference spectra were uploaded for the known fluorescing elements in the images: tyrosine hydroxylase in green (TH488), calbindin in red (CB594) and autofluorescence (Figure 5). ZEN Black then utilized the three user-identified spectra to provide three separate z-stacks parsing out the relative fluorescence contributions of tyrosine hydroxylase, calbindin and autofluorescence at every given pixel of the original image. A composite image of the tyrosine hydroxylase and calbindin channels can be created, thus effectively making an image where autofluorescence has been removed (Figure 2B).

### Sudan Black B Treatment

A total of 300 mg of (SBB; Sigma-Aldrich, 199664-25G) was left to stir overnight in 100 mL of 70% ethanol. The mixture was then vacuum-filtered to remove any undissolved SBB and the solution was allowed to rest in 4°C. Prior to use, a small volume of 0.30% SBB solution was carefully decanted and diluted to a 0.15% SBB solution.

Upon completion of the spectral unmixing protocol described previously, acetone was used to remove the nail polish sealing the coverslip. The coverslip was then carefully removed and the mounting medium was washed via pipet with PBS. The 0.15% SBB solution was pipetted drop-wise onto the tissue so as to sufficiently cover the section and was allowed to sit in the dark for 5 min. The tissue and slide were washed using a pipet with PBS. The 80% glycerol mounting medium was re-applied along with a new #1.5 coverslip and re-sealed with nail polish.

### Imaging Through “Standard” Collection (Post-sudan Black B Treatment)

The second round of imaging was performed using the same 20x/0.8 air objective on the ZEISS LSM 880 inverted confocal microscope. Image acquisition was performed using “Standard” collection mode where bandpass filters were employed to specifically collect for emissions from the 488 nm fluorophore (excited with 488 nm Argon laser, 25 mW at ∼0.7–2% power, PMT gain 640–660) between 500 and 560 nm, and the 594 nm fluorophore (excited with 594 nm HeNe laser, 2 mW at ∼15–60% power, PMT gain 665–700) between 606 and 695 nm. *Z*-stacks were obtained using identical settings as used in pre-treatment collection (1 μm steps, 8-bit, 2048 × 2048 resolution, pixel dwell of 4.12 μs, line average of 1) with a 708.5 μm by 708.5 μm FOV. Images were captured so as to encompass a nearly identical FOV as those images taken during the first round of image collection (Figure 2C). These FOVs were matched using the coordinates gathered from the first imaging session with some user-input to account for slight tissue warping as a result of SBB-treatment.

### Cell Counting

The image z-stacks (TH-UM, CB-UM, TH-SBB, and CB-SBB) were brightened in ImageJ through “Auto-Brightness/Contrast,” a feature which sets the 0.02% highest and lowest intensity pixels of each individual image as the upper and lower intensity bounds. To account for small volume differences within FOVs as a result of imaging over two different sessions, *z*-stacks of the same FOV were edited to assure that the same volumes of tissue were represented. Image files were masked from counters to blind them to each image’s treatment and TH-positive cells and CB-positive cells were counted. Multiple interrater reliability (IRR) measures based on the counts from two independent raters were calculated separately for the TH-unmixed, TH-SBB, CB-unmixed, and the CB-SBB images through a linear regression analysis. Normalized difference was calculated by subtracting the cell counts obtained from the SBB image from those of the corresponding UM image and dividing that value by the sum of the cell counts of the UM and SBB images.

### Statistics

**Figure 6:**
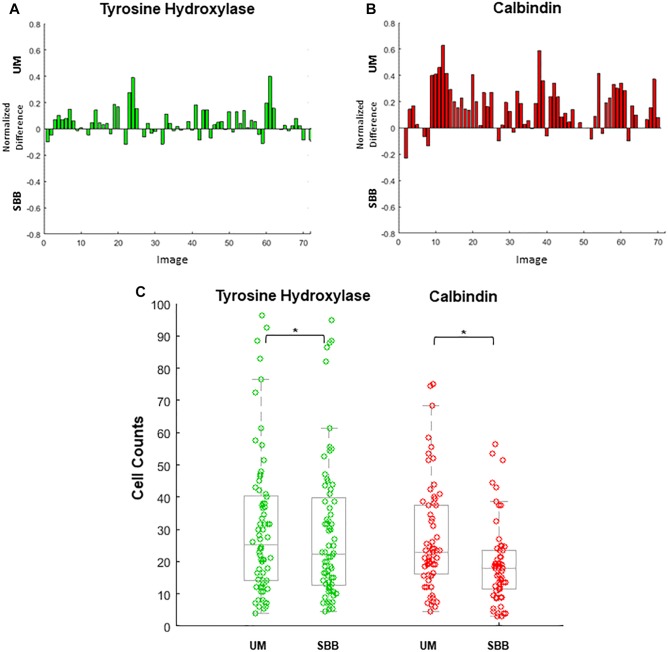
Cell numbers were counted in each image. Differences in cell counts for paired images (Unmixed (UM) and its corresponding Sudan Black B (SBB) image) were then found. **(A)** Normalized differences of tyrosine hydroxylase (TH) cell counts for UM and its paired SBB image. A positive difference value indicates a greater number of quantifiable cells in the TH-UM image compared to the TH-SBB image. **(B)** Normalized differences of calbindin (CB) cell counts for UM and its paired SBB image. A positive difference value indicates a greater number of quantifiable cells in the CB-UM image compared to the CB-SBB image. **(C)** TH (green) and CB (red) cell counts before and after treatment. In **(B,C)**, note that several CB images are removed from the count comparison because of a lack of calbindin signal. Normalized difference was calculated by subtracting the cell counts obtained from the SBB image from those of the corresponding UM image and dividing that value by the sum of the cell counts of the UM and SBB images. ^∗^ indicates *p* < 0.001.

Cell count differences between treatment conditions were analyzed separately for TH and CB using paired *t*-tests with an alpha level of *p* < 0.01 (Figure 6).

## Results

### Spectral Identification of Autofluorescence

Spectral data extracted from unstained control tissue revealed a broad emission range from 400 nm past 650 nm, with local maxima found at approximately 560 nm and 630 nm and a distinct inflection from 400 to 500 nm (Figure 4C). The emission spectrum of this autofluorescence coincides with reported data of spectrally profiled lipofuscin (Marmorstein et al., 2002). The spectral identity of this autofluorescence was then utilized in the linear unmixing protocol to classify spectral components with similar emission properties (Figure 5).

### Cell Counting

UM and SBB images of similar FOVs were “paired” in order to analyze for cell count differences as a result of treatment (Figure 6A–C). Cell counts of TH- and CB-immunopositive cells for both UM and SBB images revealed a significant reduction in the numbers of countable cells for SBB images when compared to their corresponding UM image (Figure 6A–C). TH-SBB images were found to have an average 6.99 ± 2.05% fewer cells than their TH-UM counterpart (paired *t*-test, *p* < 0.001, *t*(71) = 3.6046; Figure 6A). Furthermore, there was an average of 25.03 ± 3.40% fewer cells in CB-SBB images compared to the unmixed images (paired *t*-test, *p* < 0.001, *t*(62) = 6.7586; Figure 6B). The IRR for each of these treated groups were: TH-UM, *r* = 0.98 and TH-SBB, *r* = 0.97 where *n* = 72 images and CB-UM, *r* = 0.93 and CB-SBB, *r* = 0.57 where *n* = 63 images.

## Discussion

Neural tissue possesses intrinsic fluorescent properties that increase in abundance with age (Terman and Brunk, 2004) and can confound quantitative assessments when using fluorescence imaging techniques. The principle aim of the current study was to investigate the viability of spectral imaging and linear unmixing (UM) as an alternative to a more traditional dye-based method (SBB) of quenching autofluorescence in aged neural tissue. Two observations from this study support the conclusion that UM is a reliable and potentially more robust method of autofluorescence removal compared to SBB. First, cell count analyses revealed a significant reduction in the number of cells containing fluorescence signal after SBB-treatment compared to linear unmixing. Second, IRR measures were significantly lower in the SBB-treated calbindin images compared to the images processed with linear unmixing. This may be due to the dye’s sequestering effects on both fluorophore and background signals that make it particularly difficult for counting. Together, these two results suggest that UM is a superior method for removing autofluorescence in experiments aimed at estimating total cell numbers in neural tissue.

### Effects of Dye – Versus Imaging-Based Native Fluorescence Removal Methods on Cell Counts

As mentioned above, cell counts from two independent raters revealed a significant reduction in countable cells following SBB treatment compared to UM treatment for both TH and CB images. In the case of tyrosine hydroxylase, an average of 7% of stained cells were lost after SBB-treatment, whereas this same treatment resulted in an average loss of 25% of stained CB-positive cells. SBB likely impacted CB cell counts more than TH due to a known property of the dye in exaggerating background fluorescence in the red wavelength (Diez-Fraile et al., 2012). This added background fluorescence may reduce the relative fluorescence of the red-shifted fluorophore to make it more difficult to visualize and count. This side-effect is not observed in the green wavelength, which may account for the more modest loss of cells observed in the TH-stained images. Furthermore, it is readily apparent within our images that the TH-staining filled cells more completely than did the CB-staining, which was confined to the cell body. This differential labeling pattern of the two stains likely aided in TH’s visibility and the difficulty of resolving CB neurons after SBB-treatment.

Interrater reliability is traditionally a measure that provides an estimate of internal consistency between counters. In the present study, however, this measure was also used as an estimate of counting consistency as a result of treatment. For TH-stained images, a high IRR was observed for both UM (97.8%) and SBB (97.3%) cases that reflects high counting reliability for the relatively strong signal that this antibody emitted, regardless of treatment conditions. Interestingly, a large drop in the IRR was observed between CB-UM (93.3%) and CB-SBB (57.3%) cases. The reduction in IRR between CB-UM and CB-SBB images suggests that treatment caused enough degradation of signal in the weaker CB stain that quantification became markedly more difficult.

### Limitations of Spectral Imaging and Linear Unmixing in Native Fluorescence Removal

Spectral imaging and linear unmixing does pose disadvantages of its own. First, the procedure is only possible on microscopes equipped with hardware and software designed for “spectral unmixing” capabilities, making this option not available to some. For this experiment we had access to a ZEISS LSM 880 which was capable of spectral imaging, while linear unmixing was enabled using the proprietary ZEN Black software package. Alternative laser scanning confocal microscopes are also capable of spectral imaging and linear unmixing (e.g., Leica SP8, Olympus FV3000) using their own proprietary software (e.g., Leica LAS X for the Leica SP8 and Fluoview for Olympus FV3000).

Additionally, the spectral unmixing method requires a minimum resolution to attain the specificity necessary to decipher between emitting elements at the level of individual pixels. This necessity for greater resolution can significantly increase image acquisition and processing time, with an added disadvantage of cumbersome datasets. Also, while minimal laser powers can be used to reduce photobleaching, particularly low-intensity signals may necessitate higher laser powers and prolonged exposure that could result in significant photobleaching. Furthermore, the product of unmixing is only as good as the reference spectra that are designated as contributors to an overall image. Without sufficiently accurate reference spectra extracted from fluorescent controls, spectral components may be wrongly assigned or misinterpreted. Lastly, the overall cost of SBB is significantly lower than the overall costs accrued as a result of microscope use at imaging-core facilities.

### Methodological Considerations

While we were able to image nearly identical fields of view (FOVs) between the two (UM and SBB) imaging sessions, we purposefully did not keep pixel intensity gain settings the same between images of identical FOVs. Imaging after SBB-treatment would reliably result in significantly darker images compared to those taken during the UM imaging session due to the dye particulates obscuring some fluorescence. Thus, utilizing identical imaging settings to those used during the UM imaging session would not have been a fair comparison of SBB’s capabilities for sequestering autofluorescence and would only exaggerate some of its side-effects. We ultimately decided that it was more important to optimally visualize our stains during any particular imaging session prior to comparing treatments to each other and thus pixel intensity gain settings varied for images taken during the UM imaging session and the SBB imaging session.

In our unmixing procedure, the software utilized two, user-specified reference spectra with a third autofluorescence spectrum determined by the software that was unique to each image. We chose this approach because we determined that using the control autofluorescence spectrum from unlabeled tissue was less effective at classifying autofluorescence pixels in images of double-labeled tissue.

The scope of this particular experiment was limited to the cellular stains of TH and CB. It will be critical to specifically test the applicability of spectral imaging and linear unmixing in other preparations, including, for example, imaging at higher magnifications or using antibodies that result in punctate labeling of subcellular elements. Given that images are collected with the appropriate resolution, however, this computational method of removing autofluorescence from neural tissue should work across multiple applications.

### Summary

In this study we found that autofluorescence sequestration through SBB -treatment resulted in significantly reduced cells counts for both tyrosine hydroxylase and calbindin stains. These results provide support that spectral imaging and linear unmixing is a superior alternative to SBB -treatment, particularly in combatting autofluorescence for images with weak staining.

## Data Availability

The datasets generated for this study are available on request to the corresponding author.

## Ethics Statement

This study was carried out in accordance with guidelines stipulated in the Guide for the Care and Use of Laboratory Animals by the National Institutes of Health and all of the described protocols were reviewed and approved by the Institutional Animal Care and Use Committee at the University of California, Davis.

## Author Contributions

All authors conceived of the study. WP was involved in the processing of neural tissue for immunohistochemistry and imaging. DG provided the necessary code for image processing and statistical analyses. All authors contributed to manuscript writing and revision. CB provided funding for the work.

## Conflict of Interest Statement

The authors declare that the research was conducted in the absence of any commercial or financial relationships that could be construed as a potential conflict of interest.

## References

[B1] Aswendt M. Vogel S. Schäfer C. Jathoul A. Pule M. Hoehn M. (2019). Quantitative in vivo dual-color bioluminescence imaging in the mouse brain. *Neurophotonics* 6:025006. 10.1117/1.NPh.6.2.025006 31093514 PMC6504011

[B2] Belichenko P. V. Fedorov A. A. Dahlström A. B. (1996). Quantitative analysis of immunofluorescence and lipofuscin distribution in human cortical areas by dual-channel confocal scanning microscopy. *J. Neurosci. Methods* 69 155–161. 10.1016/S0165-0270(96)00035-0 8946318

[B3] Brizzee K. R. Ordy J. M. Kaack B. (1974). Early appearance and regional differences in intraneuronal and extraneuronal lipofuscin accumulation with age in the brain of a nonhuman primate (*Macaca mulatta*). *J. Gerontol.* 29 366–381. 10.1093/geronj/29.4.366 4209545

[B4] Clancy B. Cauller L. J. (1998). Reduction of background autofluorescence in brain sections following immersion in sodium borohydride. *J. Neurosci. Methods* 83 97–102. 10.1016/S0165-0270(98)00066-1 9765122

[B5] Dickinson M. E. Bearman G. Tille S. Lansford R. Fraser S. E. (2001). Multi-spectral imaging and linear unmixing add a whole new dimension to laser scanning fluorescence microscopy. *BioTechniques* 31 1274–1276. 10.2144/01316bt01 11768655

[B6] Diez-Fraile A. Van Hecke N. Guérin C. J. D’Herde K. (2012). “Optimizing multiple immunostaining of neural tissue,” in *Applications of Immunocytochemistry*, ed. Dehghani H. (Rijeka: InTech), 345–442. 10.5772/34588

[B7] Edwin E. E. Jackman R. (1981). Nature of autofluorescent material in cerebrocortical necrosis. *J. Neurochem.* 37 1054–1056. 10.1111/j.1471-4159.1981.tb04497.x 7320720

[B8] Eldred G. E. Katz M. L. (1988). Fluorophores of the human retinal pigment epithelium: separation and spectral characterization. *Exp. Eye Res.* 47 71–86. 10.1016/0014-4835(88)90025-5 3409988

[B9] Ershler W. Coe C. Gravenstein S. Schultz K. Klopp R. Meyer M. (1988). Aging and immunity in nonhuman primates: I. Effects of age and gender on cellular immune function in rhesus monkeys (*Macaca mulatta*). *Am. J. Primatol.* 15 181–188. 10.1002/ajp.1350150210 31968901

[B10] Feldman T. B. Yakovleva M. A. Arbukhanova P. M. Borzenok S. A. Kononikhin A. S. Popov I. A. (2015). Changes in spectral properties and composition of lipofuscin fluorophores from human-retinal-pigment epithelium with age and pathology. *Anal. Bioanal. Chem.* 407 1075–1088. 10.1007/s00216-014-8353-z 25471291

[B11] Fuxe K. (1965). Evidence for the existence of monoamine neurons in the central nervous system. *Z. Zellforsch. Mikrosk. Anat.* 65 573–596. 10.1007/BF00337069 14263017

[B12] Guzowski J. F. Timlin J. A. Roysam B. McNaughton B. L. Worley P. F. Barnes C. A. (2005). Mapping behaviorally relevant neural circuits with immediate-early gene expression. *Curr. Opin. Neurobiol.* 15 599–606. 10.1016/j.conb.2005.08.018 16150584

[B13] Hakeem A. Sandoval G. R. Jones M. Allman J. (1996). “Brain and life span in primates,” in *Handbook of the Psychology of Aging*, eds Birren J. E. Schaie K. W. (New York, NY: Academic Press), 78–104.

[B14] Jung T. Höhn A. Grune T. (2010). “Lipofuscin: detection and quantification by microscopic techniques,” in *Advanced Protocols in Oxidative Stress II. Methods in Molecular Biology (Methods and Protocols)* Vol. 594 ed. Armstrong D. (Totowa, NJ: Humana Press), 10.1007/978-1-60761-411-1_13 20072918

[B15] Kanaan N. M. Kordower J. H. Collier T. J. (2007). Age-related accumulation of Marinesco bodies and lipofuscin in rhesus monkey midbrain dopamine neurons: relevance to selective neuronal vulnerability. *J. Comp. Neurol.* 502 683–700. 10.1002/cne.21333 17436290

[B16] Kikugawa K. Beppu M. Sato A. Kasai H. (1997). Separation of multiple yellow fluorescent lipofuscin components in rat kidney and their characterization. *Mech. Ageing Dev.* 97 93–107. 10.1016/S0047-6374(97)00050-X 9226629

[B17] Lansford R. Bearman G. Fraser S. E. (2001). Resolution of multiple green fluorescent protein color variants and dyes using two-photon microscopy and imaging spectroscopy. *J. Biomed. Opt.* 6 311–318. 10.1117/1.1383780 11516321

[B18] Lichtman J. W. Conchello J. A. (2005). Fluorescence microscopy. *Nat. Methods* 2 910–919. 10.1038/nmeth817 16299476

[B19] Mansfield J. R. Gossage K. W. Hoyt C. C. Levenson R. M. (2005). Autofluorescence removal, multiplexing, and automated analysis methods for in-vivo fluorescence imaging. *J. Biomed. Opt.* 10:41207. 10.1117/1.2032458 16178631

[B20] Marmorstein A. D. Marmorstein L. Y. Sakaguchi H. Hollyfield J. G. (2002). Spectral profiling of autofluorescence associated with Lipofuscin, Bruch’s membrane and sub-RPE deopsits in normal and AMD eyes. *Invest. Ophthalmol. Vis. Sci.* 43 2435–2441. 12091448

[B21] Meister B. Askergren J. Tunevall G. Hemmings H. C. Greengard P. (1991). Identification of a dopamine- and 3′ 5′-cyclic adenosine monophosphate- regulated phosphoprotein of 32 kD (DARPP-32) in parathyroid hormone-producing cells of the human parathyroid gland. *J. Endocrinol. Invest.* 14 655–661. 10.1007/BF03347888 1663529

[B22] Mylle E. Codreanu M.-C. Boruc J. Russinova E. (2013). Emission spectra profiling of fluorescent proteins in living plant cells. *Plant Methods* 9:10. 10.1186/1746-4811-9-10 23552272 PMC3630006

[B23] Paxinos G. Huang X.-F. Toga A. W. (2000). *The Rhesus Monkey Brain in Stereotaxic Coordinates.* San Diego, CA: Academic Press.

[B24] Romijn H. J. van Uum J. F. Breedijk I. Emmering J. Radu I. Pool C. W. (1999). Double immunolabeling of neuropeptides in the human hypothalamus as analyzed by confocal laser scanning fluorescence microscopy. *J. Histochem. Cytochem.* 47 229–236. 10.1177/002215549904700211 9889258

[B25] Schnell S. A. Staines W. A. Wessendorf M. W. (1999). Reduction of lipofuscin-like autofluorescence in fluorescently labeled tissue. *J. Histochem. Cytochem.* 47 719–730. 10.1177/002215549904700601 10330448

[B26] Terman A. Brunk U. T. (2004). Lipofuscin. *Int. J. Biochem. Cell Biol.* 36 1400–1404. 10.1016/j.biocel.2003.08.009 15147719

[B27] Tigges J. Gordon T. P. McClure H. M. Hall E. C. Peters A. (1988). Survival rate and life span of rhesus monkeys at the Yerkes regional primate research center. *Am. J. Primatol.* 15 263–273. 10.1002/ajp.1350150308 31968890

[B28] Yamanaka R. Shindo Y. Hotta K. Suzuki K. Oka K. (2018). GABA-induced intracellular Mg2+ mobilization integrates and coordinates cellular information processing for the maturation of neural networks. *Curr. Biol.* 28 3984.e5–3991.e5. 10.1016/j.cub.2018.10.044 30528584

[B29] Zimmermann T. Marrison J. Hogg K. O’Toole P. (2014). Clearing up the signal: spectral imaging and linear unmixing in fluorescence microscopy. *Methods Mol. Biol.* 1075 129–148. 10.1007/978-1-60761-847-8_5 24052349

[B30] Zimmermann T. Rietdorf J. Pepperkok R. (2003). Spectral imaging and its applications in live cell microscopy. *FEBS Lett.* 546 87–92. 10.1016/S0014-5793(03)00521-0 12829241

